# Contribution of Hospital-Based Continuous Positive Airway Pressure (CPAP) Titration to the Treatment of Sleep Apnea Syndrome (SAS) Patients

**DOI:** 10.7759/cureus.74520

**Published:** 2024-11-26

**Authors:** Abir Bouhamdi, Loubna Boumekrat, Hind Bourkhime, Yassine Chefchaou, Sanae Labyad, Meryem Benjelloun, Mounia Serraj, Bouchra Amara, Mohamed Chakib Benjelloun, Mohamed El Biaze

**Affiliations:** 1 Pneumology, Hassan II University Hospital, Fez, MAR; 2 Epidemiology, Clinical Research, and Community Health, Sidi Mohamed Ben Abdellah University, Fez, MAR

**Keywords:** apnea-hypopnea index (ahi), bilevel positive airway pressure (bipap), continuous positive airway pressure (cpap), hospital titration, sleep apnea syndrome

## Abstract

Objectives: This retrospective study aims to evaluate the efficacy of continuous positive airway pressure (CPAP) therapy in treating severe obstructive sleep apnea (OSA), based on polysomnographic parameters, and to highlight the importance of hospital-based titration in optimizing treatment and guiding choices of alternative ventilation modes.

Methods: Sixty-nine patients (n=69, 100%), predominantly female (n=49, 71%), were included in the study. Polysomnographic data were collected during hospital-based CPAP titration. The efficacy of CPAP therapy was assessed through changes in sleep and respiratory parameters before and after titration.

Results: The results demonstrated significant improvements in polysomnographic parameters, including reductions in the apnea-hypopnea index (AHI), total respiratory index (TRI), and desaturation index, as well as increases in mean REM sleep time and sleep efficiency. A third of the patients (n=23, 33%) required additional titration with alternative ventilatory modes, such as BiPAP or auto-SV.

Conclusion: Hospital-based CPAP titration is essential in managing severe OSA, optimizing therapeutic outcomes, and ensuring personalized treatment. CPAP was effective in controlling nocturnal hypoventilation, reducing the need for BiPAP in many cases.

## Introduction

Severe sleep apnea syndrome (SAS) represents a significant medical challenge requiring an appropriate therapeutic approach to prevent its serious complications. Continuous positive airway pressure (CPAP), introduced in 1981, marked a major advance in the treatment of SAS and remains the reference standard today [1,2]. Recognized as revolutionary, this method relieves symptoms and reduces associated risks, thus considerably improving sleep quality and respiratory function.

Nevertheless, despite the well-documented efficacy of CPAP, its evaluation remains essential in clinical practice. Polysomnographic parameters, such as the apnea-hypopnea index (AHI) and desaturation index, provide crucial objective data to assess the efficacy of this treatment and guide therapeutic decisions. The central objective of this study is, therefore, to evaluate the efficacy of CPAP through hospital titration in the sleep laboratory under polysomnographic monitoring, in order to determine the effective pressure level (Peff), and, if necessary, to choose an alternative ventilatory mode, such as non-invasive ventilation (NIV) or self-assisted ventilation (SAV), for patients with variable responses to CPAP or specific needs. This in-depth analysis will help optimize therapeutic outcomes and ensure personalized management of patients with severe SAS, thus contributing to improved healthcare in this crucial area.

## Materials and methods

Design

This retrospective epidemiological study was conducted at the Sleep Medicine Center at Hassan II University Hospital in Fez. It included patients followed for SAS over two years, from January 1, 2022, to February 31, 2024.

Participants

Inclusion Criteria

Participants of any age followed up for SAS were included in the study, subject to presenting indications for manual CPAP titration, such as uncontrolled heart failure or rhythm disorders, combined SAS or with Cheynes-Stokes-type breathing, daytime hypoventilation (confirmed by gasometry), and obvious nocturnal hypoventilation (prolonged 5 min period of oxygen saturation below 88%).

Exclusion Criteria

Patients without respiratory comorbidity or heart failure, as well as those absent from their titration appointment, were excluded from the study.

Data collection

Clinical

Data collection involved obtaining epidemiological information such as age, sex, weight, height, and body mass index (BMI), as well as reasons for consultation and comorbidities such as heart disease, hypertension, diabetes, and others, from clinical records. In addition, each patient was administered a validated Arabic-language sleep questionnaire, including sections dedicated to assessing different aspects of sleep and sleep habits, as well as the Epworth Daytime Sleepiness Scale, which we validated in its Arabic version, with the aim of comprehensively collecting data on their sleep.

Polysomnography

Polysomnography was performed with Philips Respironics Alice 6 polysomnographs and analyzed using Sleepware G3 software.

Patients with severe SAS were given two nights: the first for polysomnographic recording and the second for CPAP titration. This titration was carried out under the supervision of a technician, gradually increasing the CPAP level until apneas, hypopneas, snoring, and micro-arousals related to respiratory events disappeared in all sleep stages and positions. In the event of persistent residual desaturation index or central events, a third night of titration was programmed under a different ventilatory mode (BiPAP or self-assisted NIV).

Statistical analysis

Data were entered into Excel and analyzed using SPSS version 26 software at the epidemiology laboratory of the Faculty of Medicine and Pharmacy of Fez, Sidi Mohamed Ben Abdellah University. Descriptive statistical analysis was performed, describing qualitative variables as percentages and quantitative data as mean ± standard deviation or median ± interquartile range (25-75). The association between CPAP and polysomnographic parameters was examined using Student's parametric test of comparison of means, with a significance threshold set at 0.05.

## Results

Description of the study population

Sixty-nine patients, ranging in age from three to 92 years (mean age 60.66±17.69 years), were included in our study, of whom 71% (n=49) were women. The most represented age group was 60-80 years, representing 48.7% (n=33) of the sample, followed by 40-59 years, representing 30.2% (n=21). The over-80 and under-40 age groups each accounted for 7% (n=5) of the sample.

The BMI was 34.78±6.44 kg/m², distributed as follows: normal (n=2, 2.89%), overweight (n=9, 13.04%), moderate obesity (n=14, 20.28%), severe obesity (n=18, 26.08%), and morbid obesity (n=7, 10.14%).

In our study, 61.4% (n=27) of patients were hypertensive, 53.8% (n=28) were cardiac, 36.5% (n=19) were diabetic, 23.1% (n=12) had dyslipidemia, 15% (n=10) suffered from gout, 8.8% (n=5) had hypothyroidism, 5.9% (n=2) had acromegaly, and 2.9% (n=2) had multinodular goiter.

In terms of reported symptoms, we noted nocturnal snoring (72.5%, n=50), excessive daytime sleepiness (34.8%, n=24) with a mean Epworth score of 11.45±6.35, chronic dyspnea (31.9%, n=22), nocturnal respiratory pauses (15.9%, n=11), and headaches (1.44%, n=1). The Epworth scale was broken down as follows: mild (n=18, 26.08%), moderate (n=15, 21.73%), and severe (n=11, 15.94%), with missing values explaining why the total did not equal 100% (Table 1).

**Table 1:** Patient data BMI, body mass index; HTA, hypertension

Variables	N (%) OR M (±SD) (N=69)
Age (years)	60.66±17.69
Gender	
Female	49 (71%)
Male	20 (29%)
BMI (Kg/m^2^)	34.78±6.44
HTA (hypertension)	
Yes	27 (61.4%)
No	17 (38.6%)
Heart disease	
Yes	28 (53.8%)
No	24 (46.2%)
Diabetes	
Yes	19 (36.5%)
No	33 (63.5%)
Acromegaly	
Yes	2 (5.9%)
No	67 (94.1%)
Nocturnal snoring	
Yes	50 (72.5%)
No	19 (27.5%)
Excessive daytime sleepiness	
Yes	24 (34.8%)
No	45 (65.2%)
Dyspnea	
Yes	22 (31.9%)
No	47 (68.1%)
Nocturnal respiratory pauses	
Yes	11 (15.9%)
No	58 (84.1%)
Epworth score	11.45±6.35

Polysomnography performed on all patients revealed a mean AHI of 54.41±25.41 events/hour, a mean RTI of 54.29±25.19 events/hour, and a mean desaturation index of 48.27±30.12 events/hour. SAS were classified according to AHI as mild (n=1, 1.44%), moderate (n=9, 13.04%), and severe (n=59, 85.5%).

The SAS phenotypes identified included SAS associated with nocturnal hypoventilation (55.5%, n=38), followed by combined SAS (14.5%, n=10) and central SAS (2.9%, n=2). Pure obstructive sleep apnea (OSA) (comprising only obstructive events) was present in 27.5% (n=19) of cases.

Quantitative univariate analysis

The results of our study revealed a significant improvement in polysomnographic parameters following treatment with CPAP.

We observed a significant reduction in the mean number of awakenings, from 22.07±13.26 (events) pre-CPAP to 17.10±8.6 (events) post-CPAP, with a mean reduction of 4.97±13.58 (events) (p=0.003). Similarly, the mean number of micro-arousals decreased significantly, from 24.40±(11.25-51.50) (events) pre-CPAP to 11.60±(4.10-20.3) (events) post-CPAP, with a mean reduction of 17.39±27.58 (events) (p=0.000).

**Figure 1:**
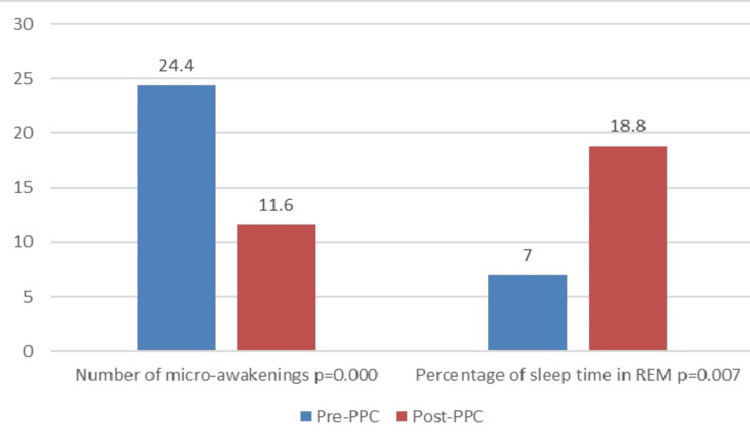
Difference in averages of sleep parameters PPC, positive pressure continuous; REM: rapid eye movement

We observed a significant increase in the percentage of mean REM sleep time relative to total sleep time, from 7±(0-14.50)% pre-CPAP to 18.8±(13-24.55)% post-CPAP, with a mean difference of 11.67±12.17% (p=0.007). In addition, mean sleep efficiency improved marginally from 68.60±15.14% pre-CPAP to 71.73±14.72% post-CPAP, with a mean difference of 3.13±17.74% (p=0.147) (Figure 1, Table 2).

**Table 2:** Mean values and differences of determined sleep parameters PPC, positive pressure continuous

Sleep parameters	Pre-PPC	Post-PPC	Difference of Post-PPC and Pre-PPC	P-value
Number of awakenings	22.07±13.26	17.10±8.6	4.97±13.58	0.003
Number of micro-awakenings	24.40±(11.25-51.50)	11.60±(4.10-20.3)	17.39±27.58	0.000
(Median ± interquartile range 25-75)	8.5±(5.45-15.65)	6.8±(3.8-12.05)	2.88±14.81	0.111
Sleep time in N1 (minutes)	58.26±14.28	53.98±19.02	4.27±19.90	0.079
(Median ± interquartile range 25-75)	14.81±12.17	16.93±12.13	-2.12±12.32	0.158
Sleep time in N2 (minutes)	7±(0-14.5)	18.8±(13-24.55)	11.67±12.17	0.007
Sleep time in N3 (minutes)	68.60±15.14	71.73±14.72	3.13±17.74	0.147

In respiratory terms, we observed a significant improvement in the AHI, from 50.7±(33.50-71.94) (events/hour) pre-CPAP to 4.20±(1-20.45) (events/hour) post-CPAP, with a mean reduction of 40.48±28.49 (events/hour) (p=0.001). In addition, the total respiratory index (TRI) decreased significantly, from 49.75±(33.42-73.42) (events/hour) pre-CPAP to 3.65±(0.72-10.82) (events/hour) post-CPAP, with a mean difference of 51.45±28.16 (events/hour) (p=0.0001).

The mean desaturation index decreased significantly from 42.70±(23.95-72.30) (events/hour) pre-CPAP to 12.4±(6.15-23.95) (events/hour) post-CPAP, with a mean difference of 29.17±27.99 (events/hour) (p=0.000).

CPAP was effective in controlling nocturnal hypoventilation, resulting in a significant improvement in mean oxygen saturation (SaO_2_), which increased by 2.34±8.8% (p=0.031) throughout sleep. Similarly, mean SaO_2_ during REM sleep increased from 86.16±8.86% before CPAP to 91.21±8.98% after CPAP, with a mean difference of -5.05±11.88% (p=0.002). There was also a reduction in the maximum duration of desaturation below 88% for 5 minutes, which fell by 2.43±23.54 minutes (p=0.039).

We also observed a significant reduction in the mean time spent below "90%" oxygen saturation, from 28.40±(9.95-52.72) minutes pre-CPAP to 3.85±(0.62-16.15) minutes post-CPAP, with a mean improvement of 23.36±30.42 minutes (p=0.0001). Maximum desaturation time showed non-significant improvement, with a mean increase of 2.43±23.54 minutes (p=0.393).

**Figure 2:**
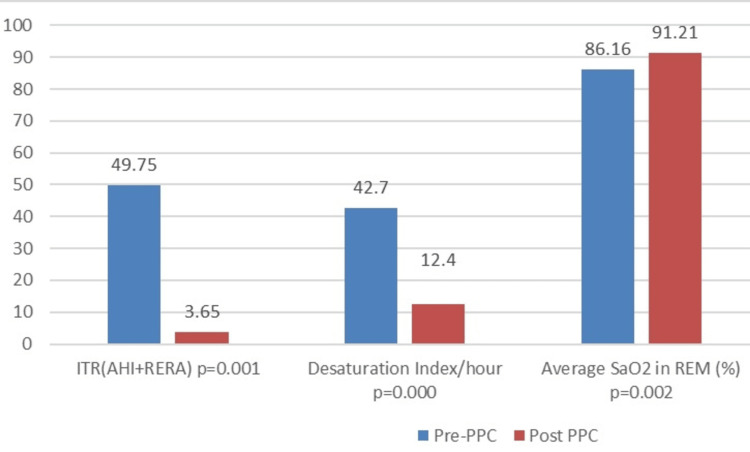
Difference in averages of breathing parameters PPC: positive pressure continuous; ITR: index of total respiratory events; AHI: apnea-hypopnea index; RERA, respiratory effort-related arousal; SaO_2_, oxygen saturation; REM, rapid eye movement

For patients with central and combined SAS, the central apnea index decreased significantly, from 0.4±(0-3.3) (events/hour) pre-CPAP to 0.2±(0-0.85) (events/hour) post-CPAP, with a mean improvement of 7.46±7.10 (events/hour) (p=0.004) (Figure 2, Table 3).

**Table 3:** Mean values and differences of determined respiration parameters PPC, positive pressure continuous; ITR, index of total respiratory events; AHI: apnea-hypopnea index; RERA: respiratory effort-related arousal; SaO_2_, oxygen saturation; REM, rapid eye movement

Breathing parameters	Pre-PPC	Post-PPC	Difference of Post-PPC and Pre-PPC	P-value
AHI	50.7±(33.50-71.94)	4.20±(1-20.45)	40.48±28.49	0.001
Median ± interquartile range, 25-75	49.75±(33.42-73.42)	3.65±(0.72-10.82)	51.45±28.16	0.0001
ITR (AHI + RERA)	42.70±(23.95-72.30)	12.4±(6.15-23.95)	29.17±27.99	0,000
Median ± interquartile range, 25-75	91.64±6.13	92.71±6.99	-1.076±9.03	0.337
Desaturation index/hour	89.57±6.03	91.91±7.09	2.34±8.8	0.031
Median ± interquartile range, 25-75	89.23±6.33	90.75±9.24	-1.52±11.08	-0.258
Mean awake SaO_2_ (%)	86.16±8.86	91.21±8.98	-5.05±11.88	0.002
Mean SaO_2_ during total sleep (%)	28.40±(9.95-52.72)	3.85±(0.62-16.15)	23.36±30.42	0.0001
Mean non-REM SaO_2_ (%)	69.55±16.05	67.12±24.63	2.43±23.54	0.0393
Mean REM SaO_2_ (%)	25.97±16.87	7.23±11.65	18.73±18.02	0.0001
Time spent sleeping below 90% (median ± interquartile range) (minutes)	17.60±(7.25-52.30)	1.60±(0.6-5.45)	21.73±24.84	0.0001
Median ± interquartile range, 25-75	14.70±(2.85-30.95)	1±(0.1-3.95)	17.46±23.10	0.0001
Maximum desaturation time below 88% in 5 min (nocturnal hypoventilation)	0.4±(0.3.3)	0.20±(0.85)	7.46±7.10	0.004

In our study, we observed that a third (33.3%, n=23) of patients required additional titration with another ventilatory mode after their CPAP treatment. Of these patients, the majority (27.53%, n=19) were titrated with a BiPAP mode, while the remainder (5.79%, n=4) were titrated with an auto-SV mode. Thus, 72.47% (n=50) of patients with nocturnal hypoventilation were treated with CPAP mode alone, without the need for NIV.

Regarding patients discharged on CPAP, 38 patients (n=38) were placed on auto-CPAP mode with the following respective expiratory pressure intervals: five patients (n=5) had Pmin at 10 cmH2O and Pmax at 14 cmH2O, four patients (n=4) had Pmin at 8 cmH2O and Pmax at 10 cmH2O, and another four patients (n=4) had Pmin at 16 cmH2O and Pmax at 18 cmH2O. The remaining patients had wider pressure intervals, with a minimum pressure of at least 4 cmH_2_O and a maximum pressure of up to 20 cmH_2_O.

In addition, eight patients (n=8) were placed on fixed CPAP with the following expiratory pressures: five patients (n=5) at 14 cmH2O, two patients (n=2) at 12 cmH2O, and one patient (n=1) at 13 cmH2O.

## Discussion

The results of our research confirm a significant improvement in polysomnographic parameters in patients suffering from severe SAS following the administration of CPAP therapy. Our study, which included 69 participants (n=69, 100%) aged from three to 92 years, (mean age: 60.66 ± 17.69 years), underlines the fact that this pathology predominantly affects an older population. This average age differs markedly from that reported in the study by Moukram et al., which documented an average age of 48 years among 306 SAS patients, suggesting demographic variations in the populations studied [3].

Our study also reveals a marked female predominance (71%, n=49), in contrast to previous observations where SAS affects more men [4,5]. However, the prevalence of SAS in women seems to increase beyond the age of 50, possibly in connection with menopause [6]. Indeed, 66% (n=32) of the women in our sample were over 50, which supports this hypothesis.

In all the cohorts examined, the prevalence of SAS increases almost linearly in adults up to the age of 65, independently of other risk factors. In men, sleep-disordered breathing (AHI>5 events/hour) is present in 7.9%, 19.7%, and 30.5% of subjects in the 20-44, 45-64, and 65-100 age subgroups, respectively. In our study, these proportions are 10%, 30%, and 40% in the same subgroups.

With regard to BMI, our sample had an average of 34.78±6.44 kg/m², significantly higher than the Wisconsin, SHHA, and Spanish cohorts, which had values of 29.9, 28.5, and 28 kg/m² respectively [4]. Polysomnography results revealed high baseline values for AHI and desaturation index, confirming the severity of SAS in this cohort of patients.

Quantitative analysis of our results revealed a significant improvement in several polysomnographic parameters following CPAP therapy. We observed a significant reduction in the mean number of awakenings and micro-awakenings, as well as an increase in mean REM sleep time and sleep efficiency. On the respiratory side, a significant reduction in AHI, TRI, and an improvement in mean oxygen saturation were recorded.

A significant finding was the diversity of individual responses to CPAP treatment, with some patients requiring titration with other ventilatory modes such as BiPAP or auto-SV. This variability underlines the importance of an individualized approach to the management of patients with severe SAS.

Studies conducted by the teams of D. Rappoport and C. Sullivan demonstrated a similar reduction in daytime PaCO2 levels under CPAP and NIV, highlighting the beneficial role of CPAP in patients who do not experience prolonged desaturation during sleep [7,8]. Subsequent works have also underscored the importance of CPAP for these patients [9,10]. Additionally, similar results have been observed in studies conducted by Spanish teams, showing significant clinical and gasometric improvements in patients treated with CPAP or NIV compared to the control group [11].

It is important to note that among the 55.5% (n=38) of OSA patients with nocturnal hypoventilation in our study, a large majority, i.e., 72.47% (n=50), were ultimately managed with a CPAP mode. Despite their nocturnal hypoventilation, these patients did not require BiPAP and showed an improvement in parameters on CPAP. These results highlight the efficacy and importance of CPAP in controlling nocturnal hypoventilation, demonstrating its crucial role in improving respiratory health and cardiovascular prognosis and reducing mortality in these patients. These findings prompt a systematic search for OSA in patients with obesity-hypoventilation syndrome (OHSS). We propose the name “AOH” (apnea obesity hypoventilation) to encourage systematic screening by sleep recording.

Persistent residual desaturation during REM sleep on CPAP may necessitate the use of NIV. Indeed, patients with SOH and SAS without hypoventilation during REM sleep generally respond well to CPAP. When hypoventilation occurs during REM sleep, this may reflect an inability of the diaphragm to compensate for the mechanical load imposed by obesity, thus necessitating NIV [12]. In our study, 27.53% (n=19) of SOH patients required NIV because of persistent desaturations on CPAP, mainly during REM sleep.

The CANPAP study assessed the impact of CPAP on mortality in heart failure patients with central SAS [13,14]. The results showed that CPAP had no influence on the rate of death or heart transplantation compared with optimal drug therapy. However, the use of SAV in the SERVE-HF study showed mixed results, with increased mortality observed in patients receiving this treatment, particularly in those with a high proportion of Cheyne-Stokes respiration and lower left ventricular ejection fraction (LVEF) [15]. In our study, 5.79% (n=4) of central or combined SAS patients required titration under auto-SV, underscoring the importance of carefully monitoring patients under this ventilatory mode.

This study has several limitations. First, it is a retrospective single-center study with a small sample size of 69 patients, which may limit the generalizability of the findings. Additionally, the study lacks long-term follow-up data to assess the sustained efficacy and potential long-term side effects of CPAP therapy.

## Conclusions

In summary, our study highlights the essential importance of hospital titration in optimizing the treatment of severe SAS. This method enables therapeutic modalities such as CPAP, BiPAP, or auto-SAS to be tailored to the specific needs of each patient. Hospital titration plays a key role in determining the definitive adoption of CPAP and in helping to parameterize other ventilatory modes. Thanks to CPAP, we were able to avoid the need for BiPAP in patients with nocturnal hypoventilation. These results underline the need for an individualized approach to improve the quality of life and respiratory health in SAS patients. Furthermore, future comparative studies of these different approaches could enrich our knowledge and guide therapeutic choices in the management of severe SAS.
